# Clinical Lecture on a Case of Epilepsy and Vertigo, in Which Bloodletting Was Employed with Advantage

**Published:** 1868-10

**Authors:** C. Handfield Jones

**Affiliations:** Physician to St. Mary’s Hospital


### CLINICAL LECTURE ON A CASE OF EPILEPSY AND VERTIGO, IN WHICH BLOODLETTING WAS EMPLOYED WITH ADVANTAGE.

By C. HANDFIELD JONES, M.D., Physician to St. Mary's Hospital.

Gentlemen: "Medio tutissimus ibis" is a wise old saw, but one which it seems not easy to act upon. The pendulum of opinion commonly deviates to one side or other of the true line. It appears to me, that at present there is some tendency to error of this kind in the case of epilepsy, which seems to be regarded by some too much as if it were invariably associated with deficient blood-supply to the brain. Very often this is the case; but that the encephalon is by no means unfrequently hyperæmic in epileptic states, and that this hyperæmia may even materially contribute to the production of the order, I think I shall be able to prove. Let me ask your attention to the following history:—

W. F., aged 49, stableman, was seen on Aug. 15th, 1865. He was sent to me by my friend Dr. Palmer, from whom I received the following statement: "Eleven years ago, he had a severe epileptic fit for the first time; he continued vertiginous, weak, and unable to work for several days; his pulse feeble and small; his skin cool and moist. Leeches, cold to the head, free purging, and iodide of potassium, failed to relieve him. He was then admitted into St. Mary's. On coming out, he told me that he was cupped and had various remedies, but all failed to do him good until he was bled, after which he rapidly and completely recovered. Since then he had several attacks of severe vertigo, with such muscular weakness as to compel him to give up work; the second was accompanied with an approach to coma. Since then they have rather diminished in severity. On each occasion, his tongue has been moderately clean; pulse between 60 and 70 decidedly soft, and not filling out the artery, without any jerk; skin pale, cool, and moist; eyes not injected; no heat of head; no thirst; appetite diminished. Each time I have tried all I could to avoid venesection, have purged freely, applied cold to the head, once cupped him, forbidden stimulants (which he says make him worse), but have each time been compelled to bleed. The last time (April 19th), I am certain that his pulse was stronger and fuller two days after the blood-letting than it had been for days before. The day after giving the iodide of potassium, he was decidedly more vertiginous." Dr. Palmer describes the patient as usually of slow, hesitating speech, and having the puzzled, hebete look of an epileptic, with a small forehead and brain-case. Such is the account given me by a most able and intelligent observer.

I subjoin the account I received from the patient's wife. She states that her husband was taken with a fit one morning at 4.45 A.M., while in bed. He was as well as ever he was in his life when he went to rest. The fit did not last long. He went to his work the same morning; but in about two hours, while at work in the mews, he was taken with another, and had seven that day, at about two hours' interval. The next day he had three fits, and continued to have them more or less frequently for three weeks until he went into St. Mary's Hospital. During this time, he lost his speech when the attacks were coming on; but, in the intervals, he was conscious and able to speak. He was leeched before he went to the hospital; but when there he was cupped and bled from the arm several times with great relief. He was there several weeks. If he got too much beer it affected his head, and made him stupid; he used to take a great deal. He had had no attack lately (Dec. 1865). So far the wife.

At my interview, I observed that his lips were pallid; his pulse 75, of good size and force; his head cool, not tender, but it felt as if it did not belong to him; his appetite was good; the tongue clean. The rhythm and sounds of the heart were normal; but the organ was rather displaced towards the epigastrium. He felt no great weakness. The giddiness he described as coming on him all at once, and lasting (till he was bled) for weeks, with intervals of comparative amendment. When the giddiness was on him he was unable to lie down, but could at other times. He had no nausea or sickness. He never passed worms, as far as he was aware. He had typhus fever when he was young; but no other illnesses, except falls—once from the roof of a house about eighty-four feet high, when he "cracked his head a bit" and was stunned for a few minutes; once also he was knocked down by a cart and struck his head.

In fact, he seems to have hurt his head a good many times. The bone was indented notably at the upper and fore part of his head towards the right side; but there was no tenderness in this situation. He had been bled at least six times, and been cupped and leeched besides. Hot weather did not try him at all, nor did cold; he could expose his bare head to the sun without any discomfort. He denied having had syphilis; and presented no outward indication of it. He was obliged to be careful in what he drank; he could not take much beer.

*August 17.* He was more giddy to-day—"just the same as a drunken man." The forehead was not hot; the eyes were dull, but not injected. Pulse quite quiet, steady, and regular. Bowels well open. Ears cool, and cheeks and prolabia actually anæmic. Pressure on the carotids caused the veins of the forehead to fill, and made the giddiness much more severe. There was no undue pulsation of the cervical or temporal arteries. The pupils were lively. His manner was very tranquil, entirely free from nervous fussiness. He was not apt to have cold feet.

I could see no rational indication for bloodletting; but, as he did not mend without, and as former experience was in its favor, I could say nothing against it. He was bled to sixteen ounces, and came to see me the next day, when he stated that he felt better in one hour after the bleeding, and was nearly well, going to work the day after. The pulse and general aspect, and condition of pupils, were just as before. I did not see him again till September 15th, when he came to see me at St. Mary's Hospital, complaining of a return of the giddiness during the last five days; it was very severe, and had not been relieved by calomel and mistura alba or by nitromuriatic acid. I ordered for him five grains of powder of colchicum thrice daily; of this he took a few doses, and got rid of his disorder completely. His pulse, I am informed, was fuller and stronger after he had taken the colchicum than it had ever been before.

*January 3, 1866.* He had been well since last date, with the exception of one attack, which was brought on by drinking, and ceased after purgation.

*December 5, 1867.* He remained quite well up to the present time. His urine was examined and found to be non-albuminous.

That the bleeding in this instance was really advantageous can, I think, hardly be questioned, regard being had not only to the immediate results, but to the subsequent condition of the patient. The attacks have not been rendered more frequent, but the reverse; in fact, for some considerable time he has been quite free from any disorder.

The vertigo must, I think, be regarded as a manifestation of epilepsy, as it succeeded the latter directly in time, and was relieved by the same measures, and by those alone, which benefited the convulsive disorder. That it depended on increased intravascular pressure can, I think, hardly be doubted, although the exterior of the head certainly exhibited no signs at all of hyperæmia. The effect of pressure on the neck, which must have told chiefly on the jugular veins as it caused distention of the frontal, was to aggravate the giddiness; and this result not only confirms the opinion above expressed, but goes to show that the blood-flow *a tergo* was free, that there was no obstruction in the arteries. The greater fulness and strength of the pulse after the bloodletting and the colchicum resulted, I believe, from the cessation of the depressing effect which the vertigo exercised on the heart itself. Although the disorder was a neurosis, it is evident that the nerve-force in this man must have been of a very different quality from that which it has manifested in sundry instances, one of which I have related in my work, p. 61, where bloodletting appeared to be the immediate cause of most severe and prolonged epilepsy.

It is probable that the injuries to the head had rendered the brain less tolerant of any undue amount of even its natural excitant, the blood; as they had also of a less natural one, the beer. If this abnormal state of excitability increased, we can well understand that depletion might be a necessary means to relieve it. The condition just referred to implies some departure from the type of healthy nutrition; and it is far from uncommon to find hyperæmia ensuing under such circumstances. It appears to me that the smaller arteries which are most concerned in hyperæmia and in like conditions go very often along with the tissue they supply in regard to their vital activity. Certainly, they are often relaxed while a tissue is vigorously performing its function; but this activity can only last for a time; and, if the hyperæmia be prolonged, the functional power of the tissue soon declines, and then the hyperæmia becomes solely injurious, and, as I wrote many years ago, "instead of supplying a want, imposes a burden." Again, if the vital power of the tissue be lowered *ab initio*, that of the arteries is very apt to be lowered along with it, and then, without any previous manifestation of functional activity, we have at once injurious hyperæmia. Of this, frost-bite and chilblains, and probably many common catarrhal inflammations, afford good examples. If we suppose this man's brain to have failed now and then in its due mode of nutrition, its nerve-power to have declined, as was not unlikely, after having been a good deal knocked about and "bemused with beer," the smaller cerebral arteries would readily fall into a like state of paresis, and thus the blood-supply would become excessive, and the nerve-power be thereby still further lowered and deranged. Bloodletting, by lessening the amount of blood, might contribute materially to enable the brain-tissue and its arteries to regain their normal condition.

As the pulse became fuller and stronger after the bloodletting and colchicum, it might have been expected that the intravascular pressure would be increased, and therewith the hyperæmia. Very possibly the pressure was increased in the radial and some other arteries; but there is no necessity that it should have been so in the carotids and vertebrals, or their smaller branches, which are the chief regulators of the blood-flow. Moreover, the very circumstance that the smaller arteries were more contracted than before, would, by lessening the facility of the escape of blood from the larger tend to produce greater fulness of the pulse, and excite the heart to stronger action.

Let me now produce to you some evidence which I have brought together from various sources, which appears to me tolerably conclusive, that active hyperæmia, determination of blood to the brain, may be the efficient cause of epileptic attacks.

C. H. Parry states that a patient, "Miss F., who has twenty or thirty epileptic fits annually, for two or three days before a fit constantly finds a constitutional enlargement of the thyroid gland increase to a very great degree; in a day or two after the fit, it returns always to its natural state, the accumulation of excitement in her subsiding at the same time." That this evident local turgescence of an organ, fed by arteries given off near those supplying the encephalon, did not cause anæmia of the latter, is tolerably clear, from its being mentioned that the head was unduly hot, the feet being cold. The same author refers to a very striking case, by which, he says, "it has been demonstrated that convulsions may depend on excessive impetus of blood in the vessels of the brain, since in that case they were removed by interrupting or diminishing the flow of blood through the carotid arteries to that organ, in consequence of which a state of sopor often ensued." In another case, mentioned by him in the same place (*Elements of Pathology and Therapeutics*, p. 254), a constant twitching of certain fibres of the flexor muscles of the forearm was uniformly suspended by compression of the carotid artery on the opposite side, while it was not diminished by compression of the artery on the same side.

Trousseau corroborates Parry's statements. He says that he has practised and recommended compression of the carotids for more than twenty years, and that he has found it highly serviceable. He makes special reference to this procedure when speaking of the convulsions attending on scarlatina dropsy; but employs it equally in other similar conditions not resulting from uræmia. He directs the artery to be compressed especially upon the side opposite to that on which the convulsions are most marked, and gives particular directions for the manipulation. He states that, when an artery is thus effectually compressed, the red flush of the face is sometimes suddenly succeeded by pallor, and that in some instances the eclamptic convulsion immediately ceases and gives place to perfect relaxation. I do not understand him to affirm that the proceeding is always successful; but, I think, none can doubt that his testimony strongly supports the position above stated. Other evidence can be cited to the same effect. In a case observed by Reimer, and alluded to by Schrœder van der Kolk (Sydenham Society's edition, p. 228), compression of the carotids succeeded twenty-two times in cutting the fit short, the patient experiencing great relief and improvement in his memory and mental condition. Labalbury (*Gazette des Hopitaux*, 1860, Sept. 15th) relates a case of puerperal eclampsia which was cured by compression of the carotids.

Parry gives the following case as a proof that epilepsy may be cured by bleeding and low diet. About thirty years ago, he states, he was sent for to see a nobleman, who had been a gross feeder but not intemperate, and had been subject to epileptic fits. He found him insensible and stertorous, after having suffered repeated paroxysms of the disorder. His face was extremely flushed, and his pulse strong, full, and laboring. His bowels had been duly open without relief. After bloodletting to fourteen or sixteen ounces, the insensibility, which had continued many hours, very quickly ceased. The bleeding was more than once repeated. Purgative and saline medicines were given. A low diet, as well as much bodily exercise, was recommended. The patient soon recovered his health, and never experienced any return of his malady. Had not the bloodletting been a right remedy in such a case as this, it is most probable it would have been injurious, and would being (repeated) have induced the speedy recurrence of the disorder, as it did in a case recorded by Dr. Copland. (*Vide* note, p. 799, art. Epilepsy.)

Dr. Parry mentions another case, which exemplifies the converse. An old man, who had lived freely, had a chronic inflammation of one of his legs accompanied by œdema. Both these affections were greatly relieved by the application of a tight bandage. In a few days he was, for the first time in his life, seized with violent epilepsy.

Graves, relating the successful treatment of a boy, aged nine, for uræmic convulsions occurring after scarlatina, by cold affusion on the head, states that, as he was sitting by the bedside of the patient, he was more than once able to predict the immediate approach of the fit, by means of watching the pulsation of the carotids, which then became much more frequent and stronger. This observation (he says), taken in connection with the fact that the pulse at the wrist became weaker and more indistinct at that very moment, suggests many interesting considerations concerning local determinations of blood. Though the uræmia was doubtless the prime cause in this instance in inducing that state of brain which gave rise to the convulsions, the peculiar hyperæsthesia of the excitable districts, yet the success of the cold affusion, leeches to the head, and purgatives, render it also, I think, clear that this perilous symptom was mainly staved off by lessening the blood-flow towards the head. So also in the convulsions which are apt to occur in the early stage of the exanthemata, there can be, I conceive, no doubt at all that the brain is, as Dr. West describes it, in a state of active congestion, true hyperæmia. Exposure to the heat of the sun may, as he says, disturb the circulation and favor congestion of the brain, and so induce convulsions or other symptoms of an overloaded state of brain, which may all subside as soon as the excited circulation has recovered its wonted balance. He gives a good case in point, very similar to one respecting which I was consulted last year, in which, after exposure to a hot sun, actual and very severe meningitis occurred.

The eclamptic attacks which occur in puerperal females are undoubtedly sometimes greatly relieved by bloodletting and antimony, and consequently can scarcely be regarded as independent of undue intravascular pressure affecting the encephalon. Of this the following case (vide *Lancet*, 1866, Nov. 3) may be cited as an example.

Mary D., aged nineteen, a well-developed, robust, plethoric woman, had always lived well, and never had a day's illness. She had one sister, aged fourteen, who some years ago was subject to occasional epileptic seizures. In her first labor, she had two fits of convulsions, in which she foamed at the mouth and bit her tongue, her features being much distorted. Mr.

Draper was sent for by the midwife in attendance, and found the patient recovering from the second attack. She was in a semi-conscious condition; face flushed; skin hot, but moist; tongue large and red, and lacerated by the teeth; head hot; pupils rather contracted; pulse 120, full, and with difficulty compressible; occasional slight muscular spasm. The child's head being at the pelvic outlet, where the midwife said it had been for about two hours and a half, the forceps were applied, and a male child delivered. It was slightly asphyxiated, but was quickly restored. After the expulsion of the placenta the patient appeared quiet and comfortable. In three-quarters of an hour the convulsions returned, the paroxysms being more severe than before. Sinapisms were applied to the nucha and the calves, and cold to the head; and a dose of calomel and jalap was given, besides the following draught every hour:

℞.—Ammon. potassio-tartrat. gr. $\frac{1}{2}$; potassæ nitratis gr. x; sodæ potassio-tartrat. gr. xx; aquæ ℥j.

Most of the powder was rejected by vomiting. After this treatment the patient remained tolerably quiet about two hours, when she had another relapse, the first being more violent and recurring more rapidly than hitherto; and she remained unconscious between the paroxysms. The face, as before, was flushed; respiration hurried and short; pulse 120 to 130, hard. As there was no albumen of consequence in the urine, about twenty-five ounces of blood were taken in a full stream from the arm. The pulse then became soft and compressible, and the patient shortly became conscious. She was ordered five grains of calomel and a minim of croton oil, and the above saline mixture to be continued. After this, the patient had no return of the fits, slept moderately well during the night, and made a good recovery.

In contrast to this case, it would be easy to cite numerous others where not depletory, but sedative treatment has been indicated, and found highly beneficial. No doubt can exist that, in puerperal eclampsia, the quality of the disorder may be different in different individuals; and, if so, why may it not be the same in epilepsy?

If any should object to the instances I have cited of uræmic, puerperal, and exanthematic convulsions being considered truly comparable to the attacks of essential epilepsy, I reply that Trousseau expressly recognizes their identity with those of epilepsy as regards the morbid phenomena, and says that they only differ in the attacks being multiple, and in the circumstances under which they occur. Neither of these features seems to me to establish any material distinction between the two classes. Multiple attacks are, we well know, met with every now and then in chronic epilepsy. As to the attendant and originating circumstances, when the visible result is so similar, it seems to me very much like begging the question at issue to affirm that the condition of brain they produce is unlike that which prevails in all cases of essential epilepsy. It is fully conceded that, among the cases of epilepsy occurring in our large towns, it is rare to meet with one where the morbid condition is not one of anæmia and weakness and undue mobility, rather than of sthenic excitement and plethora; yet I can by no means admit that the latter condition is to be excluded from our list of causes, and I believe the case I narrated at the beginning of this lecture to afford a fair instance in point. To the above evidence must be added that of Schrœder van der Kolk, to whom we are much indebted for his minute examination into the changes produced in the nervous centres by this disease. He entirely dissents from the view that suddenly induced anæmia is the essential precursory condition of the epileptic attack. Among other observations, he says: "Equally little does this view find support from the fact that, before an attack, epileptic patients are usually more excited, more lively, and more irritable, and their face and their head manifest a greater degree of congestion. In one case I was able to ascertain this with precision. In a young man aged nineteen, who, under the use of digitalis, had continued for a considerable time free from attacks, I found the heat of the head excessively great in comparison with that of the cheek," the temperature of the forehead and vertex exceeding that of the cheek by 13° Fahr. Scarcely fifteen minutes after this observation had been made, a violent attack came on. (Sydenham Society's translation, p. 299.) He has found positive advantage from the use of derivatives, issues, and setons or cupping to the neck; not only the epilepsy, but the consecutive dementia, being removed or greatly amended thereby.

I have often spoken to you of the varying quality of inflammatory disease; and I hope, from this review, you will be inclined to agree with me, that the same holds true of convulsive disorder.—*Brit. Med. Journ.*, Feb. 8, 1868.

---

The oldest Doctor in the world, Professor F. Verdugo, Salamanca, Spain, died, lately, aged 105 years. He had practised medicine for eighty years.

